# Photon-counting detector CT with iodine quantification: improved distinction between bland and neoplastic portal vein thrombosis

**DOI:** 10.1007/s00330-026-12416-8

**Published:** 2026-03-06

**Authors:** Lukas Müller, Tobias Jorg, Jan-Peter Grunz, Dirk Graafen, Aline Mähringer-Kunz, Maximilian Moos, Friedrich Foerster, Henner Huflage, Daniel Pinto dos Santos, Matteo Ligorio, Constantin Scholz, Tobias Bäuerle, Tilman Emrich, Roman Kloeckner

**Affiliations:** 1 Department of Diagnostic and Interventional Radiology, University Medical Center Mainz, Mainz, Germany; 2 Department of Radiology, University of Wisconsin, Madison, WI USA; 3 Department of Diagnostic and Interventional Radiology, University Hospital Würzburg, Würzburg, Germany; 4 Department of Internal Medicine I, University Medical Center Mainz, Mainz, Germany; 5 Department of Surgery, University of Texas Southwestern Medical Center, Dallas, TX USA; 6 Department of General, Visceral and Transplant Surgery, University Medical Center Mainz, Mainz, Germany; 7 Institute of Interventional Radiology, University Hospital of Schleswig-Holstein - Campus Lübeck, Lübeck, Germany

**Keywords:** Portal vein thrombosis, Tomography (X-ray computed), Spectral imaging, Carcinoma (hepatocellular), Iodine/analysis

## Abstract

**Objective:**

Neoplastic portal vein thrombosis (PVT) is a critical prognostic factor in hepatocellular carcinoma (HCC); however, differentiation from bland PVT remains challenging using conventional imaging criteria. Photon-counting detector CT (PCD-CT) enables quantitative iodine density (ID) assessment in every contrast-enhanced acquisition. This study evaluated the diagnostic performance of ID for distinguishing bland from neoplastic PVT.

**Materials and methods:**

In this retrospective single-center study, 104 patients with suspected PVT who underwent PCD-CT between 09/2022 and 08/2024 were included. Based on imaging, follow-up data, and multidisciplinary consensus, patients were classified into four groups: HCC with neoplastic PVT (*n* = 18), HCC with bland PVT (*n* = 29), bland PVT without malignancy (*n* = 31), and neoplastic PVT in non-HCC malignancies (*n* = 26). ID was measured in the late arterial phase (LAP) and portal venous phase (PVP) by two independent radiologists and compared with a CT feature-based score including vessel infiltration, thrombus extension, and arterial hyperenhancement.

**Results:**

ID measurements demonstrated excellent inter- and intra-rater agreement (ICC ≥ 0.99). ID was significantly higher in neoplastic PVT in both phases. Diagnostic performance was high, with sensitivities and specificities of 100% and 95.9% in LAP and 93.1% and 100% in PVP (AUC 0.98 (95% CI: 0.95–1.00) and 0.97 (95% CI: 0.92–1.00)). The feature-based score showed lower accuracy. In non-HCC malignancies, ID achieved high diagnostic accuracy in PVP.

**Conclusion:**

ID derived from PCD-CT reliably differentiates neoplastic from bland PVT in HCC and outperforms conventional CT features. In non-HCC malignancies, ID is particularly accurate in the portal venous phase, supporting its broader clinical utility as an imaging biomarker in this contrast media phase.

**Key Points:**

***Question*** *Can iodine density measured by photon-counting detector CT improve the differentiation between bland and neoplastic portal vein thrombosis?*

***Findings**** Iodine density measurements obtained with photon-counting CT accurately differentiated neoplastic from bland portal vein thrombosis and outperformed established morphologic CT features*.

***Clinical relevance**** Photon-counting CT-derived iodine density enables reliable, noninvasive identification of neoplastic portal vein thrombosis, thereby improving diagnostic confidence and treatment planning in patients with hepatocellular carcinoma and other malignancies*.

**Graphical Abstract:**

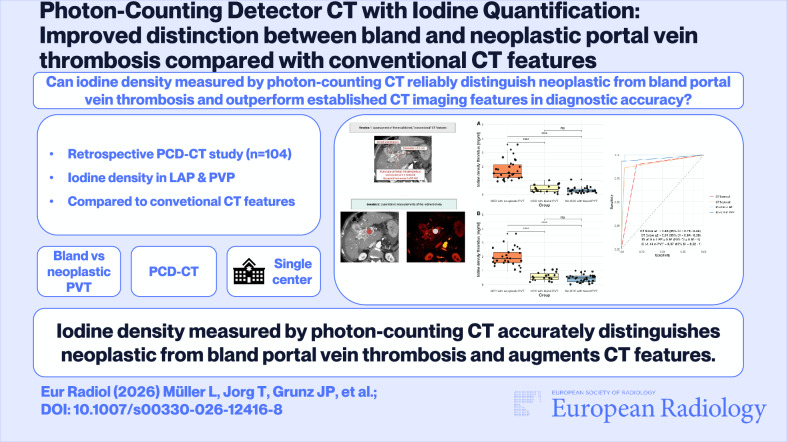

## Introduction

Hepatocellular carcinoma (HCC) is the sixth most common cancer worldwide and the third leading cause of cancer-related mortality [1]. Survival rates, however, vary considerably across tumor stages according to the Barcelona Clinic Liver Cancer classification [2–4]. A key determinant within this system is the presence of macrovascular invasion, with neoplastic portal vein thrombosis (PVT) being the most prevalent form in HCC (affecting up to 40% of patients) [3, 5]. Regardless of its extent, neoplastic PVT has significant prognostic implications and strongly influences treatment decisions [6]. Most HCCs develop in a preexisting cirrhosis. Patients with liver cirrhosis and HCC are also at high risk of developing bland PVT (with reported prevalences of up to 40%) [5, 7]. Bland and neoplastic PVT differ fundamentally in pathophysiology, treatment strategies, and prognostic significance, yet their distinction remains challenging [8]. Direct biopsy is challenging and carries inherent risks, including tumor seeding and complications related to underlying liver disease, such as an increased bleeding tendency [9]. Consequently, cross-sectional imaging is the primary diagnostic approach. While MRI provides the highest accuracy for differentiation, it is not always available or feasible [7, 10, 11]. Beyond common contraindications to MRI (such as pacemakers, other non-MRI-compatible devices, or claustrophobia), patients with liver cirrhosis frequently present with ascites, which can compromise image quality. Thus, CT plays a crucial role in the clinical management of these patients.

In addition to established CT features, such as direct vessel infiltration, thrombus extension, and arterial hyperenhancement, which are particularly valuable when assessed in combination, several review articles suggest a potential role for spectral imaging in differentiating PVT subtypes [7, 8, 12, 13]. However, iodine density (ID) as an additional imaging biomarker has only been explored in two pilot studies so far [5, 14]. These early findings indicate that ID measurements using dual-energy techniques may significantly enhance differentiation between bland and neoplastic PVT. Nevertheless, a key limitation of dual-energy imaging is that, in some systems across different vendors, the decision to acquire spectral information must be made before scanning, and certain acquisition methods are associated with higher radiation doses [15, 16]. In contrast, photon-counting detector CT (PCD-CT) provides spectral information, including ID, with every scan, thereby eliminating the need for preselection and avoiding additional radiation exposure [15, 16]. However, no study has yet investigated the potential of ID for distinguishing bland from neoplastic PVT in PCD-CT, which might be attributable to the continuously growing but still limited availability of PCD-CT scanners, including limited experience on the profile of multiphase abdominal scan protocols on this scanner type.

In addition to these technical considerations, prior dual-energy CT studies leave an important clinical question unresolved, namely, which contrast-enhanced phase is most suitable for ID-based differentiation of bland and neoplastic PVT. While Ascenti et al focused on ID measurements in the late arterial phase (LAP), Qian et al evaluated the portal venous phase (PVP) [5, 14]. Consequently, the optimal contrast phase for ID assessment remains unclear, representing a relevant gap for standardized clinical implementation.

Over the past decade, research on neoplastic PVT in malignancies beyond HCC has expanded [17]. Although less common, its prognostic impact appears equally significant in other tumor entities [17]. However, imaging in these cases is even more challenging, as CT features of PVT are less distinctive and remain underexplored. Consequently, advanced techniques are crucial for differentiating bland from neoplastic PVT.

We hypothesized that PCD-CT-derived ID can (1) differentiate neoplastic from bland PVT in HCC using absolute thresholds and (2) provide adequate sensitivity in non-HCC malignancies, particularly in the portal venous phase.

Thus, this study aimed to (1) evaluate the role of ID measurements for the distinction between bland and neoplastic PVT in patients with HCC and (2) explore the potential of ID measurements for PVT differentiation in other malignancies using PCD-CT.

## Materials and methods

This retrospective study was approved by the local ethics committee of Rhineland-Palatinate. Informed consent was waived by the ethics committee (Reg. No. 2022-16359).

### Patient selection and reference standard

From 09/2022 to 08/2024, a total of 104 patients with suspected PVT in their clinical reports of PCD-CT scans were identified using our local radiology information system.

Since histopathologic confirmation of the nature of PVT was only available in a limited number of cases, the reference standard for classification into bland versus neoplastic PVT was defined by a consensus between two independent radiologists with dedicated expertise in liver imaging (R.K., 15 years of experience; L.M., 5 years of experience). The assessment was based on an integrated evaluation of contrast-enhanced CT, follow-up imaging including MRI and ultrasound under treatment, as well as clinical, laboratory data and longitudinal clinical follow-up, as well as interdisciplinary tumor board discussions, which served as complementary background information. HCC itself was diagnosed previously by biopsy or imaging according to the classifications of the European Association for the Study of the Liver or the American Association for the Study of Liver Diseases [18, 19]. For the patients with non-HCC malignancies, biopsy served as the reference standard.

Patients were subclassified into four groups: (1) Patients with HCC and neoplastic PVT, (2) patients with HCC and concomitant bland PVT, (3) patients without any current or prior malignancy but bland PVT, and (4) patients with neoplastic PVT in a known malignancy other than HCC. Patients with concomitant bland and neoplastic thrombi were classified as having neoplastic PVT. In cases with multiple thrombi, only the one with the largest cross-sectional diameter was measured. To localize the thrombus according to its location within the PV system, the site of the thrombus was assessed (main trunk, left side, right side) as well as the extension according to the Liver Cancer Study Group of Japan classification [18]: Vp0 = no PVTT; Vp1 = segmental PV invasion; Vp2 = right anterior or posterior PV; Vp3 = right or left PV; Vp4 = main trunk and/or contra-lateral portal vein branch to the primarily involved lobe. Notably, this classification was developed for the systematic evaluation of the extent of neoplastic PVT in patients with HCC. However, for comparability, the same anatomical classification was applied across all other subgroups.

### Image acquisition

All CT scans were performed on a PCD-CT (Naeotom Alpha®, Siemens Healthineers). Consequently, the assessment of both established CT features as well as ID measurements was performed using PCD-CT data from the same scanner in a single-vendor, single-scanner setting. Scans used a 120 kVp tube voltage in single-source, multi-energy scan mode (QuantumPlus, Siemens Healthineers). All images were reconstructed with a slice thickness of 1 mm. Detailed acquisition parameters have been reported in previous publications [20–22]. Identical weight-adjusted contrast media protocols were applied for both CT types: for patients < 70 kg, 80 mL of iodine contrast agent at 3.5 mL/s; for 70–90 kg, 100 mL at 4 mL/s; and for > 90 kg, 120 mL at 4 mL/s (Ultravist® 370, Bayer Vital), resulting in iodine fluxes between 1.3 gI/s and 1.5 gI/s. Each contrast injection was followed by a 50 mL saline bolus at 3 mL/s. The hepatic arterial phase was triggered using bolus tracking in the proximal abdominal aorta, with a threshold increase of 100 HU and a post-threshold delay of 13 s. Portal venous and late phases were acquired with delays of 50 s and 180 s, respectively [22].

### Image analysis

**Fig. 1:**
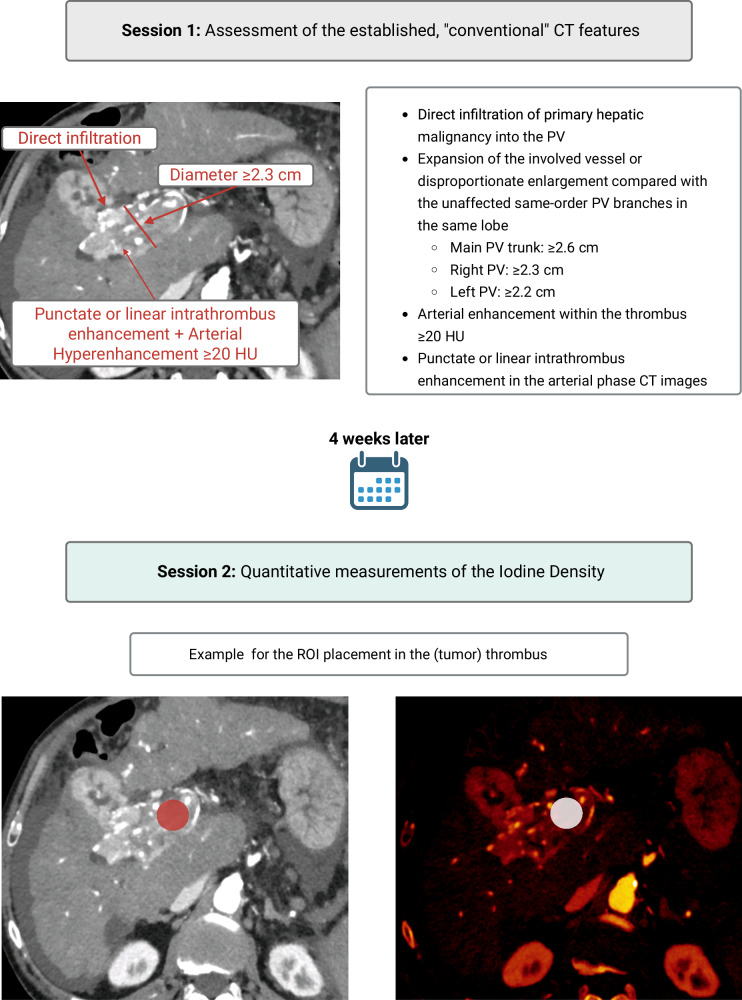
Overview of the established CT features [14] and an example of the ROI measurements. Created with BioRender. PV, portal vein

Images were independently analyzed by two board-certified radiologists with 6 and 7 years of experience in abdominal imaging (T.J. and J.P.G.) using Syngo.via (VB60, Siemens Healthineers). In an initial session, both readers jointly assessed CT features in consensus (Fig. 1). In a second session, performed 4 weeks later, each reader placed three circular regions of interest (ROIs) within the PVT on virtual monoenergetic images at 70 keV in both the LAP and PVP. ROIs were drawn with the largest feasible diameter while remaining entirely within the thrombus, thereby minimizing partial volume effects. In cases of heterogeneous thrombus composition, ROIs were preferentially positioned in areas of relatively high attenuation but were not restricted to these regions, as previously reported [14]. ROIs were then automatically propagated to the corresponding iodine maps, and the mean iodine density across the three ROIs was calculated for each reader. During ROI placement, the vessel wall and any visible artifacts were carefully avoided. In addition, to minimize the influence of interindividual hemodynamic variations, iodine density values of the thrombus were normalized to aortic enhancement in the LAP and to portal vein enhancement in the PVP. To assess intra-reader reproducibility, one radiologist reanalyzed 25 randomly selected cases in a third session conducted 4 weeks later. Representative examples of ROI placement are shown in Fig. 1.

### Statistics

All statistical analyses and graphical outputs were performed using dedicated statistical software (R, version 4.1.1, R Foundation for Statistical Computing). Categorical and binary baseline parameters are reported as absolute numbers and percentages, whereas ordinal-scaled variables are reported as medians and interquartile ranges. Normalization was performed on the aortic and portal vein enhancement by dividing the ID of the thrombus through the ID in the aorta and non-thrombotic portal vein as previously suggested [14]. Interval-scaled variables are reported as means and standard deviations (SDs). Inter-rater and intra-rater agreement were calculated using the intraclass correlation coefficient (ICC). ICC has been evaluated according to Koo and Li: below 0.5, poor; between 0.5 and 0.75, moderate; between 0.76 and 0.9, good; above 0.9, excellent [23]. Shapiro–Wilk tests were performed to assess normality. Due to the non-normal distribution of the data, Kruskal–Wallis test and Wilcoxon test were used, depending on the number of groups. Receiver operating characteristics (ROCs) were used to determine the area under the curve (AUC). Pairwise comparisons of the AUCs were performed using DeLong’s nonparametric method, which provides an asymptotically exact test for correlated ROC curves. A *p*-value < 0.05 was considered statistically significant.

## Results

A total of 104 patients were included. Mean age at time of CT was 65.5 years (SD 10.1 years), and 38 patients (36.5%) were female. Detailed baseline characteristics are provided in Table 1.

**Table 1:** Baseline characteristics

Characteristics	All patients (*n* = 104)	Group 1 (*n* = 29, 27.9%)	Group 2 (*n* = 18, 17.3%)	Group 3 (*n* = 31, 29.8%)	Group 4 (*n* = 26, 25.0%)
Sex, *n* (%)					
Male	66 (63.5)	18 (62.1)	13 (72.2)	21 (67.7)	14 (53.8)
Female	38 (36.5)	11 (37.9)	5 (27.8)	10 (32.3)	12 (46.2)
Mean age, years (SD)	65.5 (10.1)	63.2 (9.5)	68.4 (10.8)	64.7 (9.2)	67.0 (10.6)
Type of PVT, *n* (%)					
Bland	49 (47.1)	0	18 (100.0)	31 (100.0)	0
Neoplastic	55 (52.9)	29 (100.0)	0	0	26 (100.0)
Malignant disease, *n* (%)					
Yes	73 (70.2)	29 (100.0)	18 (100.0)	0	26 (100.0)
No	31 (29.8)	0	0	31 (100.0)	0
Tumor entity, *n* (%)					
HCC	47 (45.2)	29 (100.0)	18 (100.0)	0	0
Cholangiocarcinoma	15 (14.4)	0	0	0	15 (57.7)
PDAC	5 (4.8)	0	0	0	5 (33.3)
Anal carcinoma	1 (1.0)	0	0	0	1 (6.67)
Colon carcinoma	1 (1.0)	0	0	0	1 (6.67)
Gastric adenocarcinoma	1 (1.0)	0	0	0	1 (6.67)
Neuroendocrine carcinoma	1 (1.0)	0	0	0	1 (6.67)
Non-small cell lung cancer	1 (1.0)	0	0	0	1 (6.67)
Cutaneous squamous cell carcinoma	1 (1.0)	0	0	0	1 (6.67)
Liver disease, *n* (%)					
Viral	20 (19.2)	11 (37.9)	4 (22.2)	3 (9.7)	2 (7.7)
Alcoholic	34 (32.7)	10 (34.5)	10 (55.6)	11 (35.5)	3 (11.5)
MASLD	7 (6.7)	4 (13.8)	0	3 (9.7)	0
PSC	3 (2.9)	0	0	2 (6.5)	1 (3.8)
AIH	3 (2.9)	1 (3.4)	0	2 (6.5)	0
Cryptogenic	7 (6.7)	0	4 (22.2)	3 (9.7)	0
None	30 (28.9)	3 (10.3)	0	7 (22.6)	20 (76.9)
Liver cirrhosis, *n* (%)	68 (65.4)	22 (75.9)	17 (94.4)	23 (74.2)	6 (23.1)
ALBI Score					
1	11 (10.6)	4 (13.8)	1 (5.6)	4 (12.9)	2 (7.7)
2	51 (49.0)	17 (58.6)	11 (61.1)	11 (35.5)	12 (46.2)
3	42 (40.4)	8 (27.6)	6 (33.3)	16 (51.6)	12 (46.2)
Mean Charlson Morbidity Index (SD)	6.3 (3.0)	6.3 (3.0)	6.6 (2.6)	6.0 (3.0)	6.2 (2.9)
Comorbidities					
Myocardial infarction	7 (6.7)	2 (6.9)	4 (22.2)	1 (3.2)	0
Peptic ulcer disease	7 (6.7)	1 (3.4)	2 (11.1)	2 (6.5)	2 (7.7)
Diabetes mellitus	29 (27.9)	11 (37.9)	4 (22.2)	5 (16.1)	9 (34.6)
Other	17 (16.3)	5 (17.2)	1 (5.6)	3 (9.7)	8 (30.8)

Group 1: patients with HCC and neoplastic PVT; Group 2: patients with HCC and concomitant bland PVT. Group 3: patients without known current or known previous malignancy but bland PVT; Group 4: patients with neoplastic PVT in known malignancy other than HCC. Other comorbidities (all *n* < 5 in the full cohort) include: congestive heart failure (*n* = 2, 1.9%), peripheral vascular disease (*n* = 4, 3.8%), cerebrovascular accident or transient ischemic attacks (*n* = 4, 3.8%), chronic pulmonary disease (*n* = 4, 3.8%), chronic kidney disease (*n* = 3, 2.9%)

*SD* standard deviation, *HCC* hepatocellular carcinoma, *PDAC* pancreatic ductal adenocarcinoma, *PVT* portal vein thrombosis, *MASLD* metabolic associated steatotic liver disease, *PSC* primary sclerosis cholangitis, *AIH* autoimmune hepatitis

Of all patients, *n* = 29 (27.9%) were in group 1 (patients with HCC and neoplastic PVT), *n* = 18 (17.3%) in group 2 (patients with HCC and concomitant bland PVT), *n* = 31 (29.8%) in group 3 (patients without known current or known previous malignancy but bland PVT), and *n* = 26 (25.0%) in group 4 (patients with neoplastic PVT in known malignancy other than HCC).

### Iodine density for the distinction of bland and neoplastic PVT in HCC

**Fig. 2:**
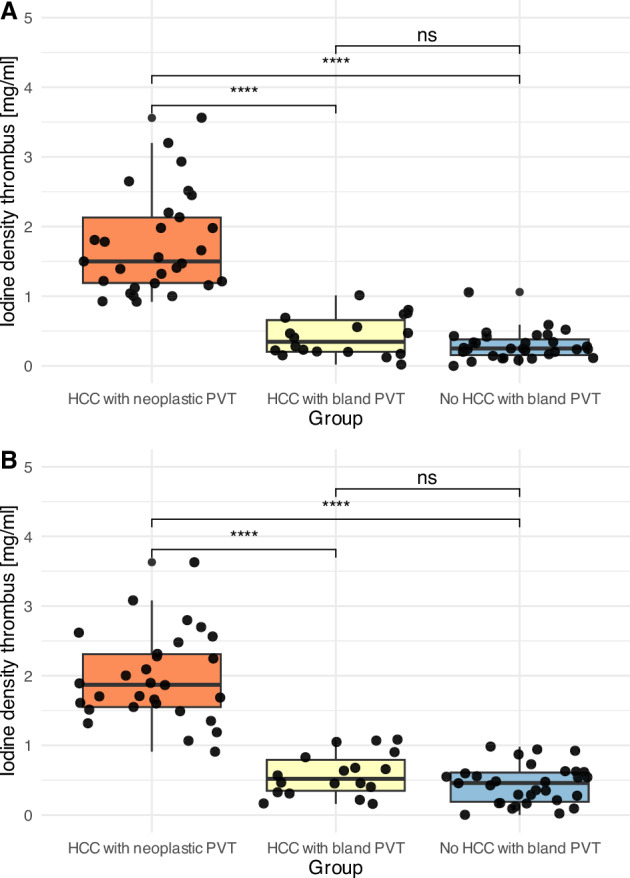
Distribution of iodine density within thrombi across three patient groups: HCC with neoplastic portal vein thrombosis (PVT, *n* = 29), HCC with bland PVT (*n* = 18), and patients without HCC but with bland PVT (*n* = 31). Boxplots illustrate the median, interquartile range, and overall distribution in the late arterial phase (**A**) and portal venous phase (**B**). Iodine density was significantly higher in neoplastic PVT compared to both bland PVT groups (**** *p* < 0.0001), while no significant difference was observed between bland PVT with and without HCC (ns, *p* ≥ 0.05). HCC, hepatocellular carcinoma; PVT, portal vein thrombosis

In LAP, the mean ID of thrombi was 1.73 mg/mL in patients with HCC and neoplastic PVT, compared to 0.42 mg/mL in those with HCC and bland PVT (*p* < 0.001). In the group of patients without tumor but with bland PVT, the mean ID was 0.29 mg/mL, and thus significantly lower than in HCC patients with neoplastic PVT (*p* < 0.001) but not significantly different from those with HCC and bland PVT (*p* = 0.18) (Fig. 2A). Normalization of ID values to the enhancement of aorta yielded a ratio of 0.16 in patients with HCC and neoplastic PVT compared to 0.04 in those with HCC and bland PVT (*p* < 0.001).

In PVP, the mean ID of thrombi was 1.96 mg/mL in patients with HCC and neoplastic PVT, compared to 0.58 mg/mL in those with HCC and bland PVT (*p* < 0.001). In the control group (patients without HCC but with bland PVT), the mean ID was 0.44 mg/mL, significantly lower than in HCC patients with neoplastic PVT (*p* < 0.001) but not significantly different from those with HCC and bland PVT (*p* = 0.13) (Fig. 2B). Normalization of ID values to the enhancement to the portal vein yielded a ratio of 0.31 in patients with HCC and neoplastic PVT compared to 0.11 in those with HCC and bland PVT (*p* < 0.001).

Using the previously suggested cut-off values of 0.9 mg/mL for LAP and 1.14 mg/mL for PVP [5, 14], this led to a sensitivity and specificity of 100%/95.9% and 93.1/100% (Table 2).

**Table 2:** Diagnostic performance of ID of the thrombus in distinguishing neoplastic (*n* = 29) from bland (*n* = 49) PVT using established cut-offs

Variable	Threshold	TP	TN	FP	FN	Sensitivity (%)	Specificity (%)
Late arterial phase							
ID thrombus	0.9 mg/mL	29	47	2	0	100	95.9
Portal venous phase							
ID thrombus	1.14 mg/mL	27	49	0	2	93.1	100

*ID* iodine density, *TP* true positives, *TN* true negatives, *FP* false positives, *FN* false negatives, *LAP* late arterial phase, *PVP* portal venous phase, *PVT* portal vein thrombosis

### Comparison to established CT features

Using contrast-enhanced CT without spectral information, sensitivity for the various established, “conventional” CT features ranged from 31% to 86% and specificity ranged from 91.8% to 95.9% (Table 3). The summation of the established features as CT score (≥ 1, ≥ 2) led to a sensitivity of 89.7% and 86.2% and to a specificity of 81.6% and 95.5%.

**Table 3:** Diagnostic performance of the CT features of the thrombus in distinguishing neoplastic (*n* = 29) from bland (*n* = 49) PVT

Variable	TP	TN	FP	FN	Sensitivity (%)	Specificity (%)
Direct infiltration	25	46	3	4	86.2	93.9
Distension	9	45	4	20	31.0	91.8
Arterial enhancement	24	46	3	5	82.8	93.9
Punctate or linear intrathrombus enhancement	15	47	2	14	51.7	95.9
CT score ≥ 1*	26	40	9	3	89.7	81.6
CT score ≥ 2*	25	47	2	4	86.2	95.9

*TP* true positives, *TN* true negatives, *FP* false positives, *FN* false negatives, *PVT* portal vein thrombosis

* Sum of the present CT features

**Fig. 3:**
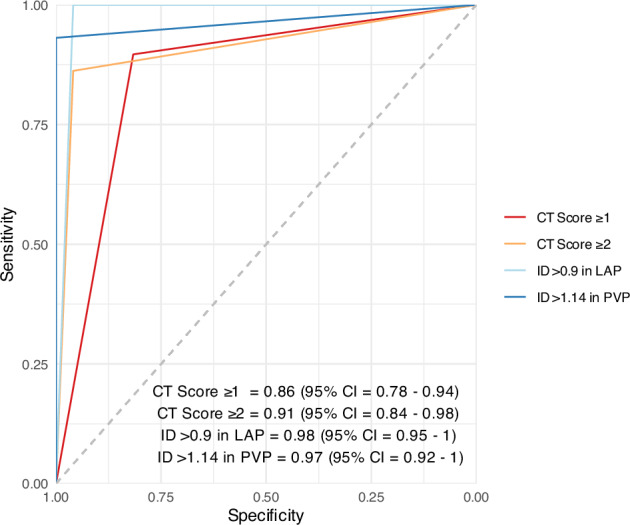
ROC curves with the corresponding AUCs for the diagnostic performance of the CT feature score with varying cut-offs ≥ 1 and ≥ 2 and ID quantification for discriminating bland and neoplastic PVT in late arterial phase (LAP) and portal venous phase (PVP). ID, iodine density

Figure 3 shows the ROC curves with corresponding AUC values for the CT score (thresholds ≥ 1 and ≥ 2) as well as ID in the LAP and PVP. Specifically, the AUC for ID in the LAP was 0.98 (95% CI: 0.95–1.00) and in the PVP 0.97 (95% CI: 0.92–1.00), whereas CT score ≥ 1 and ≥ 2 yielded lower AUC values of 0.86 (95% CI: 0.78–0.94) and 0.91 (95% CI: 0.84–0.98), respectively. Comparing the ROC curves using DeLong testing, no significant differences were observed between CT scores (*p* = 0.076) or between ID in LAP and PVP (*p* = 0.614). However, CT score ≥ 1 showed a significantly lower diagnostic performance compared with ID in LAP (*p* = 0.003) and PVP (*p* = 0.008), while CT score ≥ 2 did not differ significantly (*p* = 0.074 and *p* = 0.141, respectively). ROC analysis of the normalized ID ratios resulted in an AUC of 0.97, with an optimal cut-off value of 0.068, yielding a sensitivity of 96.6% and a specificity of 94.4%. Despite these significant differences, normalization did not further improve diagnostic performance compared with absolute iodine density measurements, as sensitivity, specificity, and AUC values remained nearly identical (Delta AUC < 0.01, *p* > 0.05).

### Iodine density in relation to the extent of PVT

In the LAP, the mean ID of thrombi in HCC with neoplastic PVT ranged from 1.59 to 2.13 across Vp1–Vp4, while in bland PVT, it ranged from 0.31 to 0.49 (all differences between neoplastic and bland PVT with a *p* < 0.01). In the PVP, ID values were 1.83–2.24 for neoplastic PVT and 0.41–0.78 for bland PVT. Except for Vp1 (*p* = 0.1, likely due to small sample size), all differences between bland and neoplastic PVT were significant (*p* < 0.01) (Supplementary Fig. 1).

### Iodine density in neoplastic PVT in known malignancy other than HCC

**Fig. 4:**
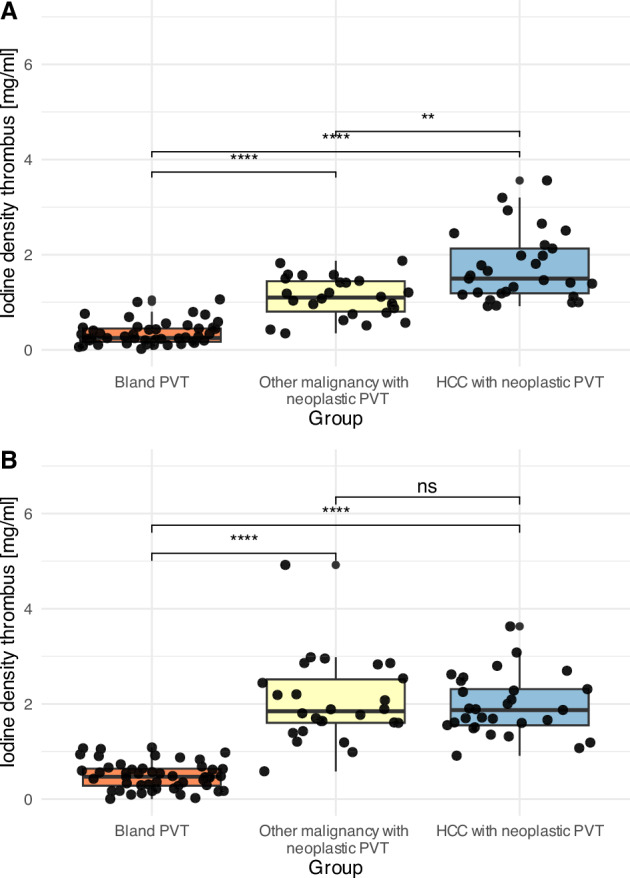
Distribution of iodine density within thrombi in patients with HCC and neoplastic PVT (*n* = 29), patients with other malignancies and neoplastic PVT (*n* = 12), and patients with bland PVT (*n* = 31). Boxplots depict the median, interquartile range, and overall distribution in the late arterial phase (**A**) and portal venous phase (**B**). Iodine density was significantly higher in neoplastic PVT of both HCC and non-HCC malignancies compared to bland PVT (**** *p* < 0.0001), while no significant difference was observed between neoplastic PVT of HCC and other malignancies (ns, *p* ≥ 0.05). HCC, hepatocellular carcinoma; PVT, portal vein thrombosis

Lastly, the distribution of ID in patients with a known malignancy other than HCC was evaluated (Fig. 4). This subgroup has a highly heterogeneous distribution underlying malignancies and should be seen as exploratory.

In the LAP, the mean ID of thrombi in patients with malignancies other than HCC was 1.11 mg/mL, compared to 0.34 mg/mL in bland PVT and 1.73 mg/mL in HCC-associated neoplastic PVT, with significant differences across all groups (*p* < 0.01). However, substantial overlap in ID distribution was observed.

In PVP, the mean ID in malignancies other than HCC was 2.05 mg/mL, compared to 0.49 mg/mL in bland PVT and 1.96 mg/mL in HCC-associated neoplastic PVT. While ID differed significantly between bland PVT and both neoplastic groups (*p* < 0.01), no significant difference was found between neoplastic thrombi in non-HCC malignancies and HCC (*p* = 0.82).

Using ID thresholds of 0.9 mg/mL in the LAP and 1.14 mg/mL in the PVP to differentiate neoplastic thrombi in non-HCC malignancies from bland PVT yielded sensitivities of 69.2% and 92.3%, and specificities of 95.9% and 100%, respectively.

### Inter-rater and intra-rater agreement

Inter-rater agreement was excellent for thrombus diameter and ID measurement for the thrombi in the LAP and PVP, with low mean bias and narrow limits of agreement (Supplementary Table 1). Intra-rater agreement for a random subset of patients (*n* = 25) was excellent across the measurements in both phases with low mean bias and narrow limits of agreement (Supplementary Table 2).

## Discussion

This study aimed to assess the value of iodine density (ID) as an additional parameter for differentiating bland from neoplastic PVT in patients with HCC. Our findings are the first to demonstrate the diagnostic advantage of measuring ID using PCD-CT, achieving higher accuracy than established imaging features. Additionally, this is the first study to investigate ID in non-HCC neoplastic PVT. In these patients, ID in PVP yielded high sensitivity and specificity in the differentiation of neoplastic and bland PVT.

So far, two pilot studies have investigated the role of ID in detecting neoplastic PVT in patients with HCC using dual-energy CT [5, 14]. Notably, both studies used systems requiring preselection of dual-energy mode, whereas in PCD-CT, as applied in this study, spectral information is inherently available with every scan. The study by Qian et al measured ID in the PVP in 44 patients with macroscopic PVT, including 29 with HCC [14]. Neoplastic PVT showed a significantly larger vessel diameter (1.61 vs. 1.25 cm) and higher ID (2.67 vs. 0.92 mg/mL), with ID outperforming the same CT feature-based diagnostic score used in our study in sensitivity (100% vs. 88.9%) and specificity (91.7% vs. 83.3%). Applying the same ID cut-off of 1.14 mg/mL, ID achieved comparable sensitivity (93.1%) and specificity (100%) in our study. However, Qian et al did not assess the predictive value of individual CT features separately. Ascenti et al investigated LAP ID in 34 patients with HCC [5]. Unlike Qian et al, their reference standard required at least two CT features indicative of malignancy. Neoplastic thrombi were larger (3.7 vs. 2.0 cm), and CT features had a sensitivity of 92.3% and specificity of 85.7%, consistent with our findings. Their ID cut-off of 0.9 mg/mL yielded a sensitivity of 100% and a specificity of 95.2%, closely aligning with our results (100% sensitivity, 95.9% specificity). Additionally, inter-rater agreement was near-perfect (96%) with a mean difference of 0.1 mg/mL, consistent with our findings. Overall, these studies support the diagnostic value of ID in PVT differentiation and highlight the potential benefit of combining ID with established CT features.

Notably, in patients with non-HCC malignancies, the sensitivity of ID in the LAP (69%) was markedly lower than that in the PVP (92%) and than the LAP sensitivity observed in HCC (96%). A plausible explanation lies in the differing enhancement characteristics: non-HCC malignancies frequently exhibit enhancement patterns resembling those of their respective primary tumors and are therefore often hypodense relative to the surrounding liver parenchyma, whereas HCC typically demonstrates strong arterial hyperenhancement. Nevertheless, because non-HCC lesions represent solid structures with at least partial contrast uptake, ID tends to increase during the portal venous phase, which likely accounts for the improved sensitivity in this setting. Thus, in non-HCC malignancies, ID derived from the PVP should be preferred over LAP-based measurements, given the lower sensitivity observed for LAP in this cohort.

In addition to the absolute diagnostic performance of ID, our ROC analysis provides further insights into its relative value compared with conventional CT features: Both LAP and PVP phases yielded excellent AUC values (0.98 and 0.97, respectively), which were significantly higher than those of the conventional CT score ≥ 1 and numerically superior to the CT score ≥ 2. While no significant difference was observed between LAP and PVP, the slightly higher AUC in LAP may reflect the characteristic arterial hyperenhancement of HCC-related thrombi, whereas the consistently strong performance of PVP underscores its robustness across tumor types. The finding that ID outperformed conventional features in a direct comparison emphasizes its potential as an independent and reliable biomarker. Taken together, these findings suggest that ID quantification can provide diagnostic accuracy at least equivalent to, and in many cases exceeding, established multiparametric CT features, thereby supporting the diagnostic pathway in patients with suspected PVT.

Although ID was normalized to vascular enhancement (aorta in the LAP and portal vein in the PVP) to account for potential hemodynamic variability, this adjustment did not further improve sensitivity, specificity, or overall diagnostic performance. These findings underscore the robustness of absolute iodine density measurements, which appear largely independent of systemic perfusion differences.

To date, this is the first study confirming the use of ID acquired with PCD-CT. Clinically, this is highly relevant, as conventional dual-energy CT requires preselection of spectral imaging on some vendor platforms and may involve higher radiation doses, depending on the acquisition method [15, 16]. In contrast, PCD-CT provides spectral information, including ID, with every scan, facilitating broader clinical adoption. Additionally, PCD-CT offers advantages such as improved contrast-to-noise ratio, reduced electronic noise, preservation of low-energy photons, and enhanced spatial resolution, further strengthening its diagnostic utility [24].

Accurate identification of neoplastic PVT has direct therapeutic implications, as its presence constitutes a decisive factor in the BCLC classification [3], precluding curative options such as resection or transplantation and guiding the choice toward systemic or locoregional treatments. Reliable PCD-CT-based differentiation of bland versus neoplastic thrombi may therefore improve treatment allocation and patient outcomes. In our HCC cohort, ID-based assessment resulted in the correct identification of approximately three additional patients with neoplastic PVT who would have been misclassified using conventional imaging criteria alone. As the presence of neoplastic PVT represents a defining factor for advanced disease within the BCLC classification, these cases would have been at risk of inappropriate downstaging and potential consideration for curative treatment options such as resection or transplantation. At the same time, the high specificity of ID indicates that only very few patients would be falsely classified as having neoplastic PVT, thereby minimizing the risk of unjustified exclusion from curative treatment pathways. In non-HCC malignancies, accurate diagnosis of neoplastic PVT can equally affect therapy selection, for instance, supporting systemic therapy initiation or, in selected cases, making radiotherapy a more relevant treatment option.

Direct comparative studies between PCD-CT and DECT for the differentiation of bland and neoplastic PVT are currently not available and were also not part of this study. However, our study shows that reported sensitivity and specificity values of conventional imaging parameters for the detection of neoplastic thrombus, as well as ID, are generally comparable to the studies of Ascenti et al and Qian et al [5, 14]. PCD-CT might potentially provide specific advantages in more subtle or smaller portal vein tumor thrombi (Vp1/2) due to its higher spatial resolution and has the benefit of inherent spectral information available in every scan.

To date, no study has examined ID measurements across different thrombus extents. In our study, ID performed consistently across all anatomic locations based on the Japan classification, suggesting high usability independent of PVT extent.

This study has several limitations. First, its retrospective, single-scanner, single-center design may limit generalizability and, as a direct consequence, yields relatively wide 95% confidence intervals for the AUC estimates, reflecting the limited sample size and the associated uncertainty in discriminative performance. Furthermore, inherent differences between the four patient groups in terms of underlying liver disease, the presence and etiology of cirrhosis, and tumor-related factors are inevitable given the clinical nature of these entities. While these variations reflect real-world patient heterogeneity, they may have influenced iodine density measurements and should be taken into account in future, prospectively balanced studies. Although the sample size is larger than in previous studies, variations in imaging protocols across centers require further investigation. Additionally, the retrospective identification of patients based on PCD-CT reports may have introduced selection bias, as small or ambiguous portal vein thrombi that were not explicitly mentioned in the original radiology reports could have been missed. Consequently, our cohort may overrepresent more conspicuous cases of portal vein thrombosis, which should be taken into account when interpreting the reported diagnostic performance. All scans were performed on a single PCD-CT scanner with scan parameters and uniform contrast media injection and timing. While these scan parameters were based on published standards [20–22], variations in scanner technology or acquisition protocols at other institutions may affect iodine density measurements and thus influence reproducibility. External validation under different technical conditions will therefore be important to confirm the generalizability of our findings. Second, histopathologic correlation was available in only a few cases, as direct biopsy in these patients is rare and carries significant risks [9]. This limitation implies that our (partially subjective) reference standard primarily relied on imaging features, follow-up, and interdisciplinary consensus, which may introduce a risk of misclassification in individual cases and further limit the independent applicability of the sensitivity and specificity values reported. Nevertheless, this reflects real-world clinical practice, where noninvasive imaging is the accepted diagnostic standard in HCC according to current guidelines [4]. While ID enhances diagnostic reliability, it does not replace established imaging criteria, though, in our study, ID alone performed as well as the combined approach. Third, all scans were obtained from a single scanner, without intermodal comparison. Future studies should evaluate different imaging modalities, including various dual-energy CT techniques and PCD-CT. Notably, previously established ID cut-offs were reproducible in our study. Fourth, ROIs were placed in the most enhancing thrombus region, consistent with prior studies [5, 14]. While smaller ROIs may introduce variability, inter- and intra-rater agreement was high across all thrombus sizes. Fifth, hemodynamic variations (e.g., cardiac output and volume status) can influence ID measurements. We included patients with various underlying etiologies of liver disease, which might have a different influence on hepatic hemodynamics, which were not taken into account in our study design. Further studies on the influence of different causes of liver disease on hepatic hemodynamics are needed. Despite this, ID demonstrated high diagnostic accuracy in our study and in prior dual-energy CT reports. Normalization to aortic and portal vein enhancement could improve inter-subject comparison, though Qian et al noted that this method is suboptimal, as it does not fully account for the interplay between arterial and portal blood supplies [14]. Further research is needed to refine this approach. Sixth, in our study, the administration of contrast medium was weight-adjusted, which may have influenced the measurement of ID. Furthermore, we did not include the delayed phase in our analysis, as it may be subject to institution-specific variability and not available for non-HCC entities, whereas LAP and PVP are more consistently standardized across centers. Last, the accessibility of PCD-CT is limited at the moment (e.g., through higher costs), which might restrict the widespread adoption. With the growing availability of scanners, our results need to be validated in detail.

## Conclusion

The findings of this study underscore the potential of ID as an additional imaging biomarker for differentiating bland from neoplastic PVT in patients with HCC. In both, the late arterial and portal venous phases, ID demonstrated higher accuracy than established imaging features and showed high inter-rater reliability. In non-HCC neoplastic PVT, ID achieved high accuracy in the portal venous phase, suggesting that this phase should be prioritized for evaluation in these patients.

## Supplementary information

ELECTRONIC SUPPLEMENTARY MATERIAL

## Abbreviations

AUC: Area under the curve
HCC: Hepatocellular carcinoma
ICC: Intraclass correlation coefficient
LAP: Late arterial phase
PCD-CT: Photon-counting detector CT
PVP: Portal venous phase
PVT: Portal vein thrombosis
ROC: Receiver operating characteristics
ROI: Regions of interest
