# Supplementary material for: Photon-counting detector CT with iodine quantification: improved distinction between bland and neoplastic portal vein thrombosis
Source: Eur Radiol. 2026 Mar 6;36(7):5380–91. doi: 10.1007/s00330-026-12416-8 (PMC13282226; doi:10.1007/s00330-026-12416-8)
Supplement: Supplementary file 1 — ELECTRONIC SUPPLEMENTARY MATERIAL [file 330_2026_12416_MOESM1_ESM.pdf]

Photon-Counting Detector CT with Iodine Quantification: Improved distinction between bland and neoplastic portal vein thrombosis compared with conventional CT features

ELECTRONIC SUPPLEMENTARY MATERIAL

Supplementary figures:

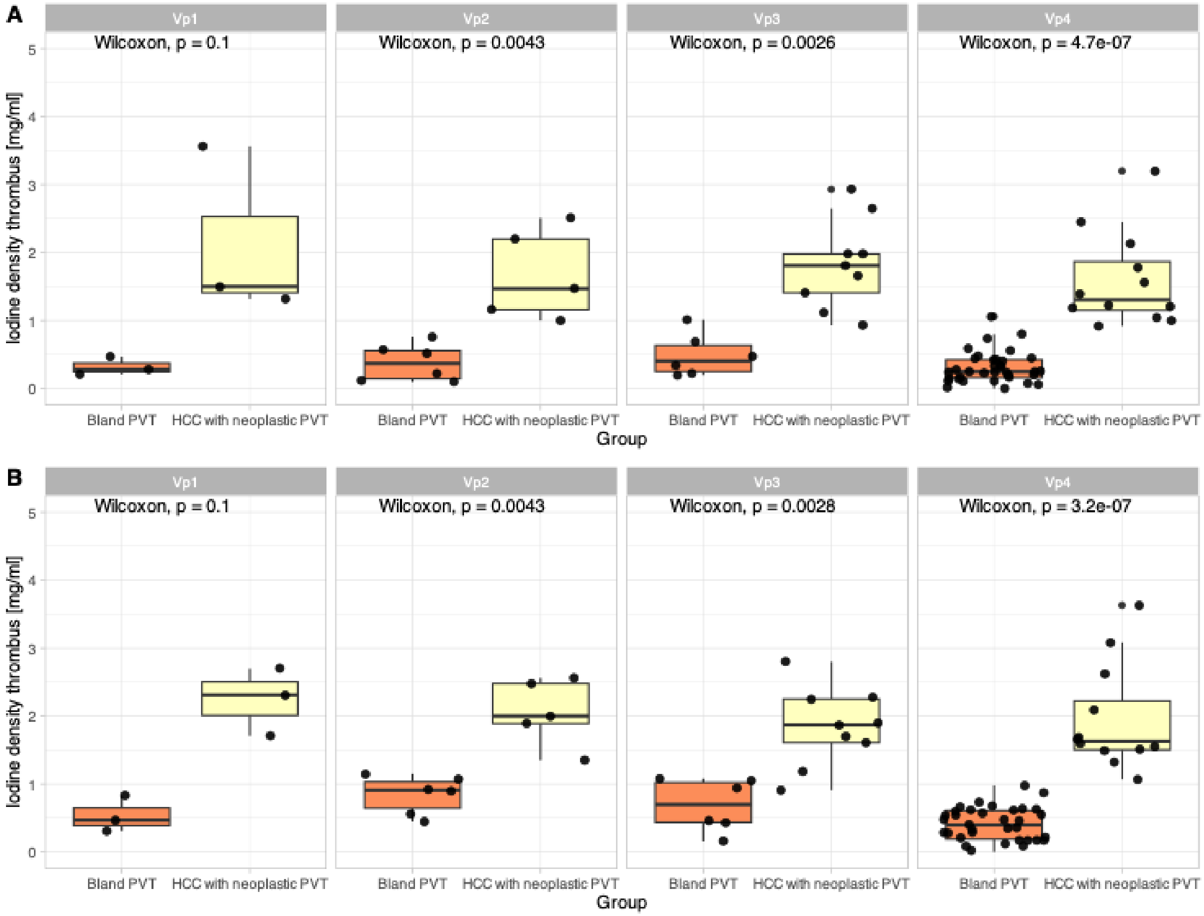

Supplementary Figure 1. Comparison of ID between bland and neoplastic PVT for the different stages of PVT extent according to the Japan Classification

Supplementary tables:

Supplementary Table 1. Inter-rater agreement: Intraclass correlation coefficient and mean bias with limits of agreement in late arterial and portal venous phase

	ICC (95% CI)	Mean bias (± 1.96 SD)
Thrombus diameter	0.99 (0.98 – 0.99)	0.03 (-0.19, 0.24)
Late arterial phase		
ID thrombus	0.99 (0.99 – 1.00)	0.01 (-0.14, 0.17)
Portal venous phase		
ID thrombus	0.99 (0.99 – 0.99)	-0.01 (-0.26, 0.25)

Supplementary Table 2. Intra-rater agreement: Intraclass correlation coefficient and mean bias with limits of agreement across the ID measurements in arterial and portal venous phase

	ICC (95% CI)	Mean bias (± 1.96 SD)
Thrombus diameter	0.99 (0.97 – 0.99)	0 (-0.22, 0.21)
Late arterial phase		
ID thrombus	1.00 (0.99 – 1.00)	0 (-0.10, 0.11)
Portal venous phase		
ID thrombus	1.00 (0.99 – 1.00)	0 (-0.12, 0.13)
